# Cartilage Injuries and Posttraumatic Osteoarthritis in the Wrist: A Review

**DOI:** 10.1177/19476035211021909

**Published:** 2021-06-15

**Authors:** Jonny K. Andersson, Elisabet Hagert, Mats Brittberg

**Affiliations:** 1 Department of Surgery, Aspetar Orthopaedic and Sports Medicine Hospital, Doha, Qatar; 2 Department of Orthopaedics, Institute of Clinical Sciences, The Sahlgrenska Academy, University of Gothenburg, Göteborg, Sweden; 3 Arcademy, H.M. Queen Sophia Hospital, Stockholm, Sweden; 4 Department of Clinical Science and Education, Karolinska Institutet, Stockholm, Sweden; 5 Department of Health Promotion Science, Musculoskeletal and Sports Injury Epidemiology Center, Sophiahemmet University, Stockholm, Sweden; 6 Cartilage Research Unit, Region Halland Orthopaedics, Kungsbacka Hospital, University of Gothenburg, Kungsbacka, Sweden

**Keywords:** wrist, cartilage repair, chondral injury, degenerative arthritis wrist, arthroscopy

## Abstract

**Objective:**

Focal cartilage injuries, and posttraumatic osteoarthritis (OA) in the wrist are likely common and a cause of wrist pain. To estimate the incidence of cartilage lesions and to understand the pathomechanisms leading to wrist cartilage injuries and OA, a literature review on the subject was performed combined with a presentation of one of the authors’ own experience.

**Design:**

This study includes a literature review of the topic. As a comparison to the review findings, the observations of one of the authors’ consecutive 48 wrist arthroscopies, were assessed. PubMed, Scholar, and Cochrane databases were searched using the keywords “cartilage injury AND wrist AND treatment” and “wrist AND cartilage AND chondral AND osteochondral AND degenerative OA.”

**:Result:**

A total of 11 articles, including 9 concerning chondral and osteochondral repair and treatment and 2 regarding posttraumatic OA, were retrieved. The cartilage repair treatments used in these articles were drilling, osteochondral autograft, juvenile articular cartilage allograft, and chondrocyte implantation. One article displayed concomitant cartilage injuries in displaced distal radius fractures in 32% of the patients. The review of our findings from a 1-year cohort of wrist arthroscopies showed 17% cartilage injuries.

**Conclusion:**

There is a lack of knowledge in current literature on cartilage injuries and treatment, as well as posttraumatic OA in the wrist. Cartilage injuries appear to be common, being found in 17% to 32% of all wrist arthroscopies after trauma, but no guidelines regarding conservative or surgical treatment can be recommended at the moment. Larger prospective comparative studies are needed.

## Introduction

Focal chondral and osteochondral injuries in the wrist are likely common and a cause of wrist pain.^1^ Those injuries could be divided into 3 groups related to injuries of ligaments, fractures, and the vascular supply.

### Ligament Injuries

**Figure 1.**
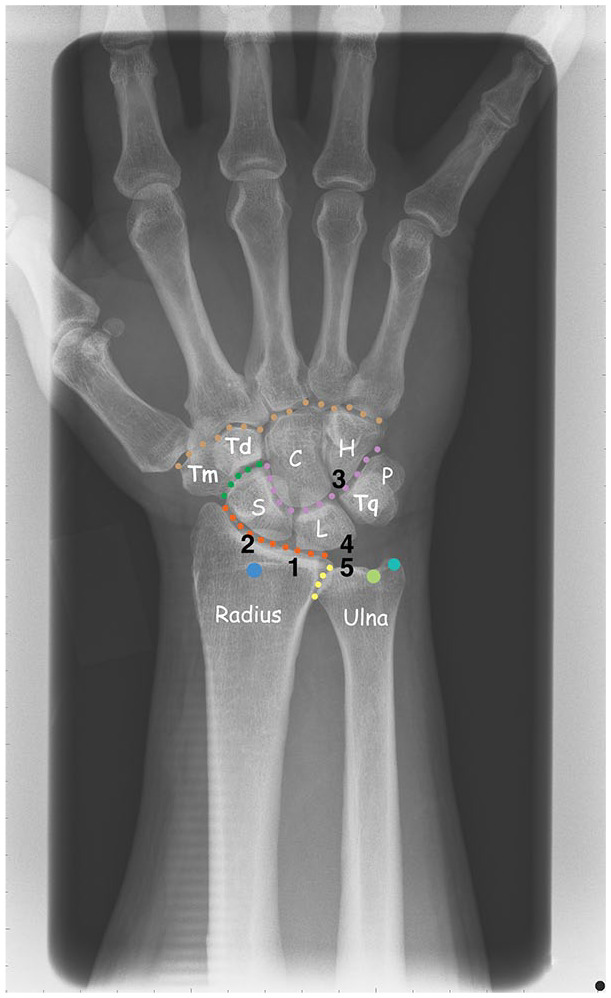
The bones and joints of the wrist. Numerals 1 to 5 display the most common described locations of chondral injuries in the wrist (S = Scaphoid, L = Lunate, Tq = Triquete, P = Pisiforme, Tm = Trapezium, Td = Trapezoid, C = Capitate, H = Hamate).

**Figure 2.**
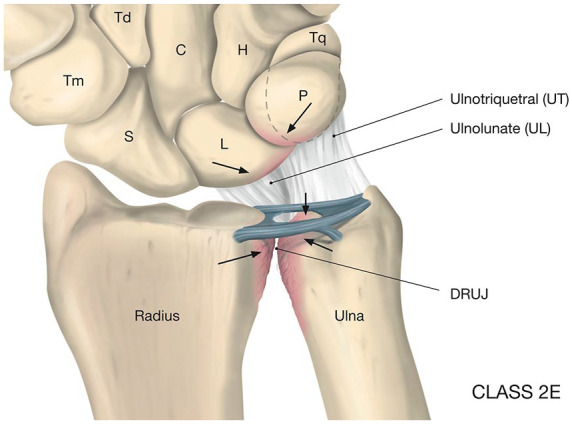
The gradual development of OA in ulnar impaction. The cause is an ulnar positive variance (ulna longer than radius) giving increased load of the ulnar aspects of the wrist, beginning with a chondral lesion at the ulnar-proximal part of the lunate and the radial ulnar proximal part of the hamate (HALT), as well as a central part of the triangular fibrocartilage complex (TFCC), followed by chondral impact at the ulnar head, finally ending up with degenerative OA in the distal radio-ulnar joint (DRUJ).

The wrist joint connects the ends of the radius and ulna with 8 carpal bones (the scaphoid, lunate, triquetrum, pisiform, trapezium, trapezoid, capitate, and hamate), which are stabilized by 24 ligaments (**Fig. 1**).^2^ Subsequently, chondral and osteochondral injuries and secondary development of osteoarthritis (OA) are often concomitant with ligament injuries, such as:

1. Scapholunate ligament injuries (SL)—leading to development to an advanced scapholunate collapse (SLAC) and a gradually increasing development of OA.^3^
2. Lunotriqeutral injuries (LT) and the associated chondral injury; hamate arthrosis lunotriquetral ligament tear (HALT).^4,5^
3. Injuries to the triangular fibrocartilage complex (TFCC), Palmer Type 1.^6^
4. Degenerative TFCC lesions (Palmer class 2) all involve the central portion and are staged from A to E, depending on the presence or absence of a TFCC perforation, lunate and ulnar chondromalacia, lunotriquetral ligament perforation, or degenerative radiocarpal arthritis. Besides in connection with ligament injuries, those lesions could more be seen in connection with fractures such as in secondary ulnar impaction,^7,8^ with chondral injury at the head of the ulna and the opposing surface of the lunate (**Fig. 2**).

### Fractures

**Figure 3.**
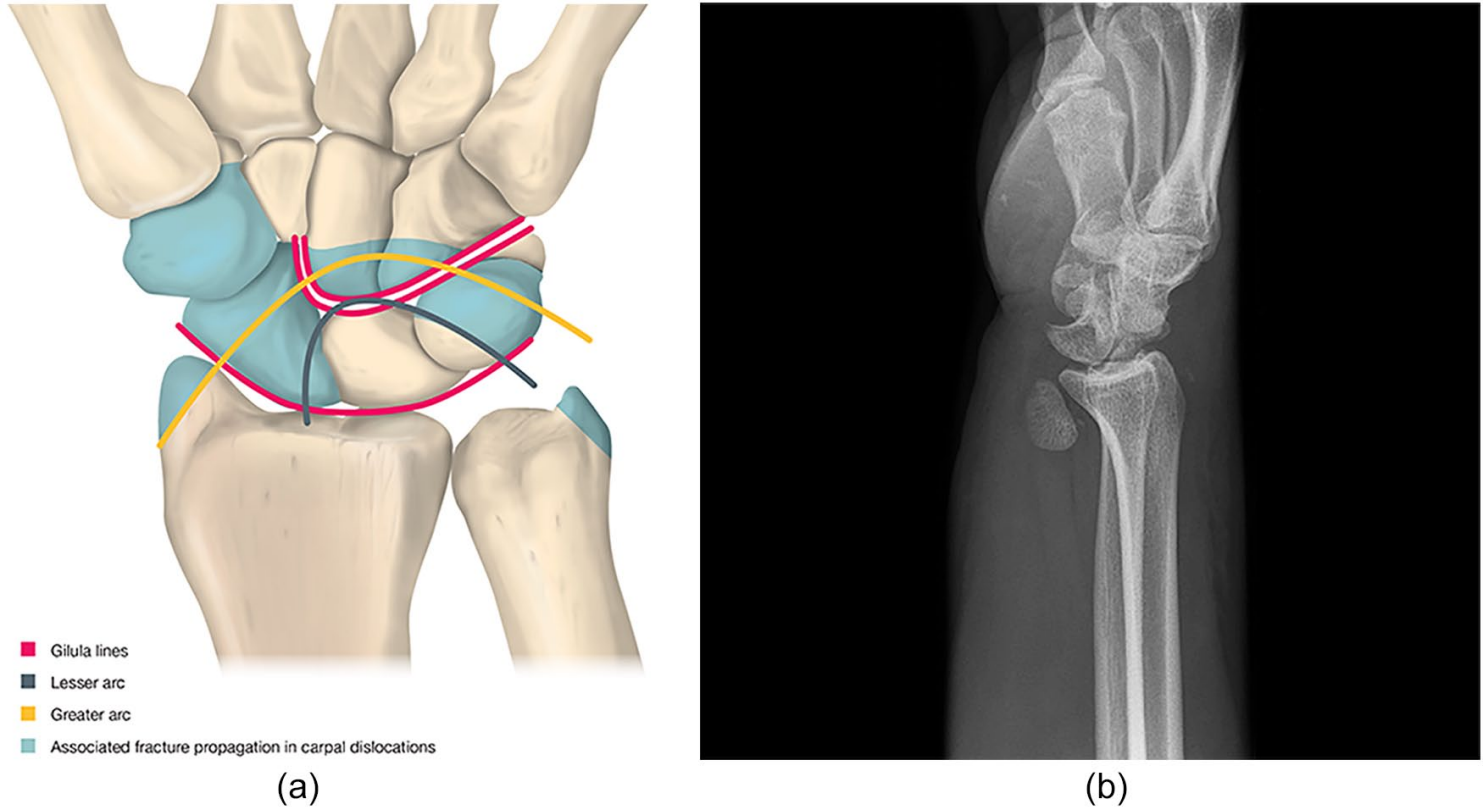
(**a**) Schematic presentation of typical patterns of perilunate dislocations and transscaphoid perilunate fracture dislocations. Normal Gilula’s lines are also displayed. (**b**) Perilunar dislocation X-ray, AP view could almost be normal. The diagnosis is obvious in the lateral view.

1. Posttraumatic chondral lesions after carpal bone fractures
2. Fractures of the radial styloid, the scaphoid, the trapezium, the capitate, and the triquetrum could all be associated with dorsal or volar perilunate dislocations and subsequent cartilage damage (**Fig. 3a** and **b**)
3. Gradually increasing development of OA is seen after nontreated scaphoid fractures with nonunion (SNAC)

A majority of those carpal fractures involves the scaphoid bone.^9^

### Vascular Cause

Injuries to the vascular supply of the carpal bones may result in osteonecrosis and collapse of the cartilage surface with subsequent OA development. The causes of the vascular disruptions could be multifactorial but a massive single impact or repeated micro-trauma could be causative in conditions such as

1. Preiser’s disease (avascular necrosis of the scaphoid bone). Mainly of unknown origin but injuries to the nutrient artery through trauma could lead to ischemia throughout the scaphoid with bone collapse and OA development.^10^
2. Kienböcks disease (avascular necrosis of the lunate bone). Disruption of the vascular supply induces osteonecrosis and overlying collapse of cartilage layer developing into OA. Trauma involvement is common.^11^

**Figure 4.**
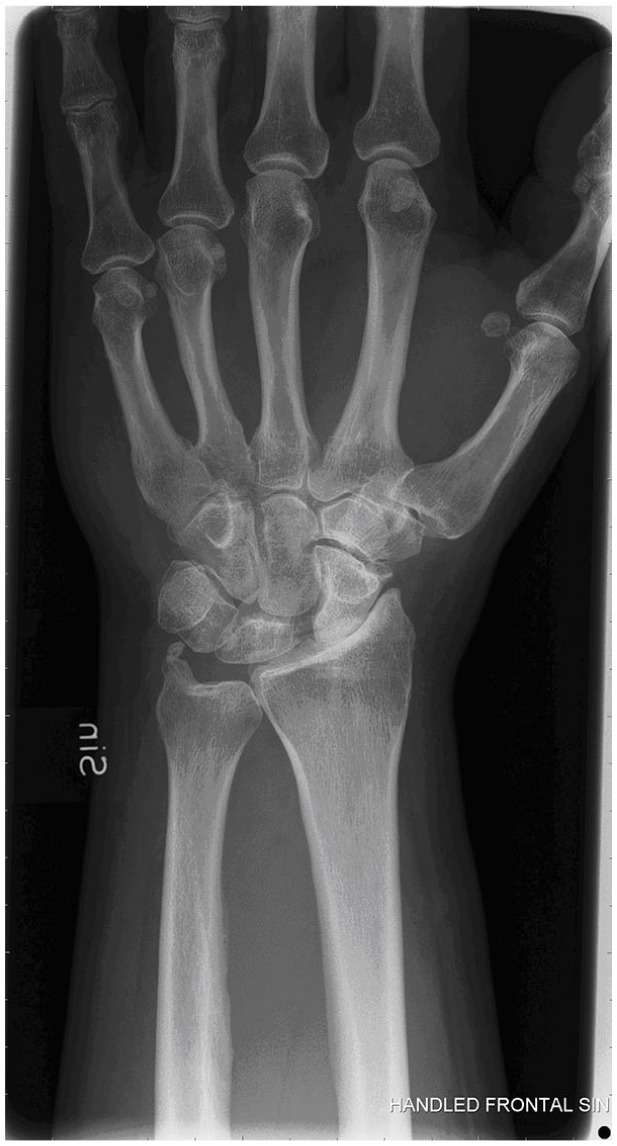
Scapholunate advanced collapse after an SL injury, with subsequent arthritic changes at the RC and MC joints (SLAC III).

**Figure 5.**
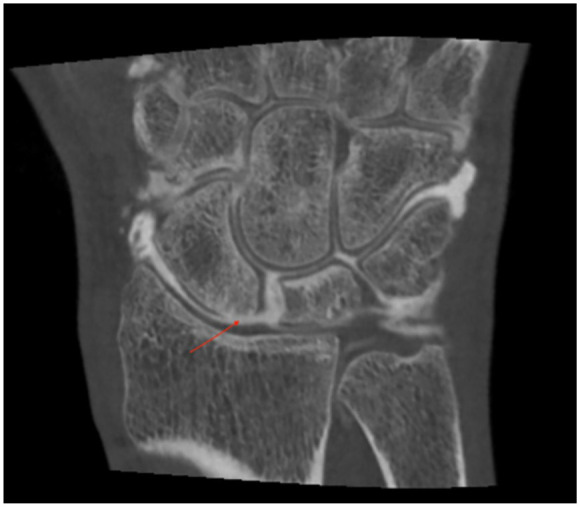
The use of intraarticular contrast fluid and cone-beam computer tomography (CBCT) can reveal even small cartilage injuries. The arrow shows the cartilage injury proximal at the scaphoid. Contrast is seen in the SL and LT spaces caused by SLAC.

To what extent the various ligament injuries with concomitant cartilage injuries and solitary cartilage injuries progress into OA is not known (**Figs. 4** and **5**). There is still no evidence that different wrist injury treatments will alter such a development. Furthermore, there is still no consensus in terms of the treatment of cartilage injuries in the wrist. While microfracturing and methods of cartilage repair and transplantation have been described and used in the clinical setting at hand surgery departments, the treatments have only been presented by other authors in a limited number of patients, as case reports or at scientific meetings.^12,13^

This is a review of the reported existence of cartilage lesions and posttraumatic OA found in the wrist after trauma in combination with one of the authors’ (JKA) cartilage lesion findings in 48 wrist arthroscopies, performed during 1 year, evaluated in 2019.

The purpose of the study was to analyze the current treatment options of cartilage injuries in the wrist and the current knowledge found in the literature in this field of interest. The aims were to find out how common these injuries are and if any specific treatment is superior to others, as well as assessing the possible gradually development to posttraumatic OA.

## Methods

### Literature Search Strategies: Cartilage Injuries and Posttraumatic Degenerative OA

A systematic review according to PRISMA^14^ was not possible, due to the limited number of articles, with only level of evidence (LoE) IV-V and heterogeneous follow-up assessments.

The broad search strategy “cartilage injury AND wrist” in PubMed, Scholar, and Cochrane’s databases was performed on February 2, 2021, and revealed 906 articles. More directed and focused search strategies (“cartilage injury AND wrist AND treatment” and “wrist AND cartilage AND chondral AND osteochondral AND degenerative OA”) were performed the same day. “Chondral AND injury hand wrist” revealed 23 articles, “cartilage injury AND wrist AND treatment” 26 articles, and “osteochondral injuries AND hand wrist” 78 articles.

Among those 78 hits, articles with irrelevant topic for this article were excluded (*n* = 52), leaving a total of 26 articles for primary inclusion. After exclusion of all reviews, any duplicates, and non-English studies, 9 articles could finally be included in terms of cartilage injuries.

Data extraction included categorizing the articles in the diagnostics and frequency of chondral and osteochondral injuries and a description of different methods of treating these injuries in the wrist. The search strategies in Scholar and Cochrane did not add any articles compared with PubMed, but only duplicates.

Thus, 26 (*n* = 9 after excluding the others because of irrelevant aim) related to chondral repair and treatment in the wrist were found. This should be compared with 9,670 articles found dealing with chondral and osteochondral injuries in the knee where, among those, the majority were of significant relevance. Level of evidence (LoE), according to Sackett,^15^ of the articles was also analyzed.

In terms of posttraumatic OA the following search strategies were used:

Wrist AND cartilage AND chondral AND osteochondral AND OA AND degenerative AND repair AND subchondral (*n* = 0)
Wrist AND cartilage AND chondral AND osteochondral AND degenerative OA (*n* = 15, but all excluded due to irrelevant aim)
Wrist AND cartilage AND chondral AND osteochondral AND post traumatic arthritis (*n* = 0)
Wrist AND cartilage AND chondral AND osteochondral AND OA (*n* = 9, but all irrelevant aim)
Wrist AND cartilage AND chondral AND osteochondral AND osteoarthrosis (*n* = 3, 1 excluded because of irrelevant aim, included: *n* = 2)^13,16^
Wrist AND cartilage AND chondral AND osteochondral AND degenerative OA/osteoarthrosis (*n* = 0)
Wrist AND degenerative arthritis (*n* = 1825, after exclusion, *n* = 1, that also describes the treatment).^16^ Rheumatoid arthritis was the main subjects in these found articles.
Wrist AND degenerative OA (*n* = 258, *n* = 9 after exclusion for irrelevant topic, after exclusion for irrelevant topic; *n* = 2).^12,16^ Thus, only 2 articles were included in terms of wrist and OA. Articles of salvage procedures in wrist OA^17^ was also discussed in the Discussion section.

Articles about nontraumatic midcarpal instability (carpal instability non-dissociative, CIND) as a cause of OA, as well as cartilage injuries and degenerative arthritis in the basal thumb and the scaphotrapeziotrapezoidal joint were excluded as the focus of this review was on pure wrist trauma.

**Figure 6.**
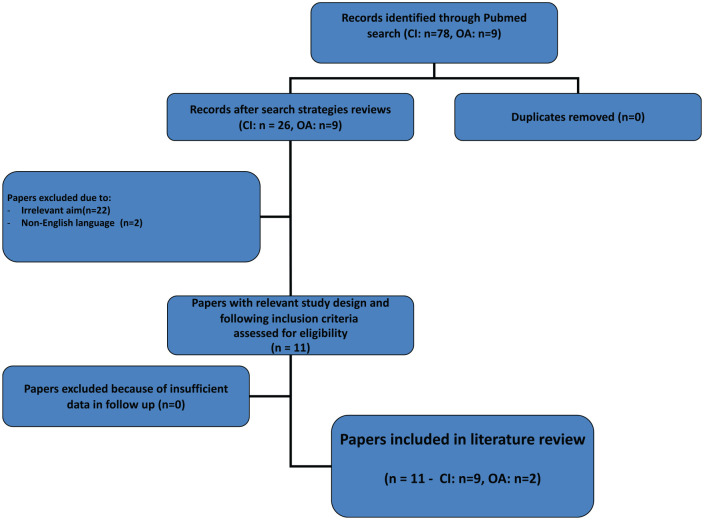
Search strategies (CI = cartilage injuries, OA = osteoarthrosis).

The search strategies are displayed in **Figure 6**.

### Wrist Arthroscopies

An assessment of a single surgeon’s experience of 48 arthroscopies (JKA), performed during 1 year—assessed in 2019—in terms of the incidence of diagnosed chondral and osteochondral injuries was performed. The results of this review and assessment serve as indicator of a possible comparison with former published papers in this area of interest.

### Ethics and Statistics

Ethical approval was performed by the University of Gothenburg, in terms of the review of the author’s performed wrist arthroscopies (Dnr: 977-1). Informed consent was approved by all participants.

Data are presented as numeric values and no statistical analysis was used.

## Results

### Literature Review

The search strategies “cartilage injury AND wrist” in PubMed, Scholar, and Cochrane databases revealed 906 articles, and the search strategies “cartilage injury AND wrist AND treatment” displayed 26 articles.

The treatments used in these articles were arthroscopic drilling, osteochondral autograft (13), and/or juvenile articular cartilage allograft (12).

Only LoE IV-V articles were found.

### Diagnosis and Evaluation

The bones and joints of the wrist are shown in **Figure 1**, as well as the most commonly described localizations of chondral and osteochondral injuries.

#### Lesion Classification

To better describe the appearance of wrist cartilage injuries, Culp *et al*.^18^ have provided a modified Outerbridge classification,^19^ with Grade I representing softening of the hyaline surface, Grade II consisting of fibrillation and fissuring, Grade III representing a fibrillated lesion of varying depth in the articular surface, and Grade IV having a full-thickness defect down to bone. Culp *et al*. suggested that Grades I to III lesions are treated with debridement and synovectomy. Grade IV lesions can be treated with abrasion chondroplasty and subchondral drilling. The International Cartilage Regeneration and Joint Preservation Society (ICRS) classification has never been used in research on the wrist, but as it describes the depth of cartilage lesions one may consider use of this classification as well.^20^

#### Wrist Imaging

In order to adequately diagnose lesions that appear after trauma, imaging is of great importance. Magnetic resonance imaging (MRI) is not reliable enough to disclose wrist ligament injuries and cartilage injuries.^21^ The use of intraarticular contrast fluid and cone-beam computer tomography (CBCT) can reveal even small cartilage injuries and is a promising new diagnostic tool in these cases (**Fig. 5**). CBCT arthrograms^22^ have been shown to have a slightly higher specificity than magnetic resonance arthrography. The sensitivity of CBCT arthrography is also better for cartilage lesions, except for those on the chondral surface of the lunate, according to Suojärvi *et al*.^23^ Suojärvi *et al*. showed that 10/21 patients with wrist pain had cartilage injuries (47, 6%), indicating that cartilage injuries may be far more prevalent than previously documented.

On regular radiographs, minor triquetral or ulnar-sided lunate sclerosis or cyst formation may be signs of ulnocarpal wear. Few studies have been conducted to investigate the use of MRI for diagnosing the etiology of wrist pain. MRI has not been shown to be sufficient enough to disclose ligament or cartilage injuries,^21^ as the sensitivity, specificity, positive predicted value, and negative predicted value are too low. In cases of rheumatoid arthritis affecting the wrist joint, MRI has revealed that cartilage damage progression is preceded by osteitis and synovitis, but is most influenced by preexisting cartilage damage suggesting primacy of the cartilage damage pathway in certain patients.^24^ No other studies were found in our search strategies in terms of comparison between the different imaging methods to diagnose solitary cartilage injuries.

The use of intraarticular contrast fluid and CBCT can reveal even small cartilage injuries (**Fig. 5**) and have higher accuracy than MRI in terms of chondral injuries in the wrist.^21,22^

#### Anatomical Variations

There are 2 types of the carpal bone lunate.^25^ In type II lunate a facet exists on the medial side of the lunate to be in articulation with the proximal pole of the hamate. Type I lunate lacks such a facet. Type II lunates have different biomechanical behaviors with a higher risk of developing degenerative changes in the hamato-lunate joint causing ulnar-sided wrist pain.

The scaphoid is the most prominent bone of the carpal bones and its motion pattern in relation to other carpal bones is important to know about when to understand injury mechanisms. Variations in scaphoid motion secondary to lunate morphology may contribute to the development of STT OA (scaphoid-trapezium-trapezoid arthritis).^26^

### Diagnostic Studies: Cartilage Injuries

#### Cartilage Injuries in Relation to Distal Radius Fractures

Focal chondral and osteochondral injuries following intraarticular distal radius fractures are common.^1^ Cottias *et al*.^27^ described in 1997 that intraarticular distal radial fractures with a step of more than 2 mm are prone to lead to focal cartilage lesion and subsequently development of OA. They also concluded that focal cartilage lesions are probably a common cause of chronic wrist pain.

The incidence of associated cartilage and ligament lesions in distal radial fractures in young adults were evaluated arthroscopically by Lindau *et al*.^1^ Fifty initially displaced fractures were evaluated. Cartilage lesions were found in 16 patients (32%). The authors concluded that cartilage lesions were rather common and may explain poor outcomes after well-healed distal radial fractures. Subchondral hematomas—without any significant focal cartilage injury—in distal radial fractures does not lead to the early onset of OA. However, an inferior outcome 1 year after surgery, compared with patients without fractures, was seen, according to Mrkonjic *et al*.^28^

Forty-two patients who underwent open reduction and internal fixation of distal radial fractures were arthroscopically evaluated for SL and TFCC injuries with or without chondral surface damages in a study by Swart and Tang.^29^ Forty-five percent of the patients had SL injuries, 50% had TFCC injuries, and 29% had articular cartilage injuries.

Tarabin *et al*.^30^ showed that posttraumatic radiocarpal articular cartilage damage did not differ between fractures with intraarticular and extra-articular extensions, but patients with fractures had notably higher risk of articular cartilage degradation compared with healthy controls.

#### Cartilage Injuries and Carpal Bone Fractures

##### Scaphoid bone

Caloia *et al*.^31^ found that 15 of 24 patients with acute scaphoid fractures had associated ligamentous and/or chondral/osteochondral injuries. They concluded that fluoroscopy during the placement of a compression screw may decrease the rate of subchondral penetration, that may happen in as much as approximately 20% of the patients.^31^

Scaphoid impaction injuries occur in sports involving a forced and repetitive hyperextension of the wrist, where the dorsal lip of the radius is forced into contact with the proximal/dorsal articular surfaces of the scaphoid. These injuries are primarily seen in weightlifters and gymnasts. If conservative treatment fails, a dorsal scaphoid ridge or dorsal radial lip cheilectomy has most often been performed. If the procedure is unsuccessful in relieving pain, a coexisting cartilage defect may be the source of pain.^32,33^

##### Midcarpal bones

Dautel and Merle^34^ showed that chondral defects and/or arthritic lesions of the ulnar portion of the midcarpal joints were observed more often in wrists with type II lunates (2 joint facets articulating with the capitate and hamate). Cartilage lesions of the midcarpal joints in type I lunates (1 joint facet) were always associated with other ligamentous and/or osteochondral lesions, whereas the same lesions could be found isolated in type II lunates. The observed association of lesions appeared to point to a specific trauma and specific anatomical variations as causes of some cartilage lesions. However, Viegas^35^ showed no pathologic conditions of the hamate bone (HALT) in type I lunates.

##### Iatrogenic cartilage injuries

Iatrogenic cartilage penetration injuries especially after compression screw fixation of scaphoid fracture and nonunion have also been described.^36^

#### Posttraumatic Osteoarthritis

Wrist OA is usually secondary to posttraumatic events and most often develops in the joints that involve the scaphoid.^3^

Several general causes of wrist OA are listed below:

• Senescence
• Gender—women are more likely to develop OA^37^
• Obesity
• Previous wrist injuries
• Genetic predisposition^38,39^
• Kienböck’s disease^40^
• Preiser’s disease^41^
• Rheumatoid arthritis
• Postinfectious arthritis

The search strategies in PubMed in terms of OA displayed 2 articles, apart from the duplicates found in the search strategies regarding wrist cartilage/chondral injuries. No conclusion could be stated in terms of the incidence of secondary development of posttraumatic OA, after solitary primary cartilage injuries.

Laulan *et al*.^16^ published a review (LoE V) in 2015 describing the different types of OA in the wrist, mainly after ligament injuries and scaphoid nonunion, ending up in SLAC and SNAC wrist, and the proper treatment options available. There are, however, several articles describing more specific ligament injuries and subsequent OA development and treatments. Weiss and Rodner point out that chronic scapholunate tears are known to produce intercarpal instability with an altered kinematics and joint loading, and subsequent development of joint degeneration of the radiocarpal joint.^42^

Weiss and Rodner also pointed out common causes of wrist post-traumatic OA:

- Scapho-lunate advanced collapse (SLAC)
- Scaphoid fracture nonunion collapse (SNAC)
- OA secondary to an intraarticular fracture of the distal radius or ulna, or from an extra-articular fracture resulting in malunion and abnormal joint loading

However, Weiss and Rodner did no not describe any percentages of trauma types involved in the development of wrist OA.^42^

Repetitive use of the wrist has been discussed as a risk factor for OA development and may be seen as microtrauma of the joint. However, strong and moderate evidence exists for no increased risk of hand and wrist OA related to highly repetitive tasks.^43^

The possible development of OA after solitary cartilage injuries is not proven in studies, apart from in intraarticular distal radius fractures and iatrogenic cartilage penetration injuries after compression screw fixation of scaphoid fractures and nonunions.^36^

To summarize, osteoarthritis in the wrist is most commonly a result of untreated SL injuries and scaphoid nonunions,^3^ where the injuries lead to carpal instability (carpal instability dissociative, CID), carpal collapse, and finally scapholunate advanced collapse (SLAC) or scaphoid nonunion advanced collapse (SNAC) wrist (**Fig. 4**).

Regarding frequency, high-energy injuries, in particular perilunate and transscaphoid perilunate fracture dislocations (**Fig. 3a** and **b**), are associated with a mean incidence of posttraumatic osteoarthritis of almost 40% (range, 7% to 92%).^44,45^ Signs of posttraumatic OA after perilunate dislocations and trans-scaphoid perilunate fracture dislocations increase progressively, but some patients have minor symptoms at follow-up after more than 10 years.^44,45^ However, approximately 30% of these patients are not able to return to heavy manual work.^44,45^

### Treatment of Local Cartilage Defects and OA

**Figure 7.**
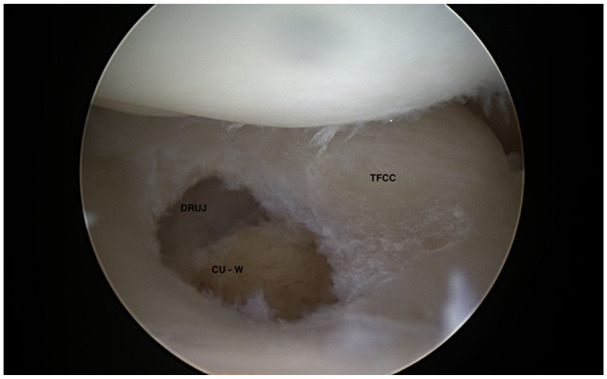
Arthroscopic Wafer osteotomy in ulnar impaction (CU-W = Caput ulna after Wafer osteotomy).

**Figure 8.**
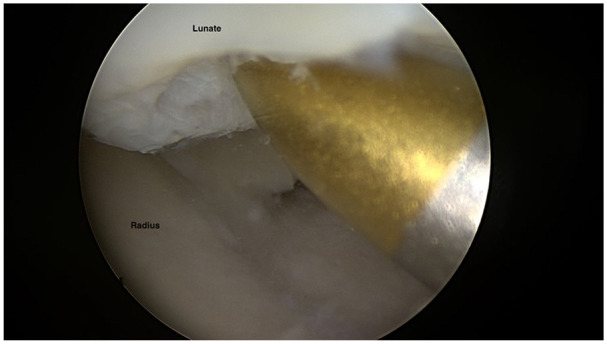
Microfracture of a proximal ulnar chondral injury at lunate.

**Figures 9.**
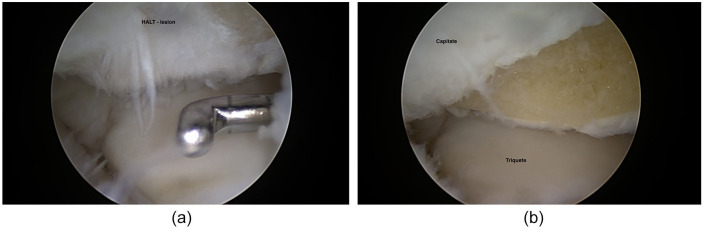
HALT lesion at proximal-radial part on hamate (**a**), treated by shaving and abrasion (**b**).

**Figures 7** to **9** display common arthroscopic treatment options of wrist cartilage injuries.

#### Chondroplasty

The use of radiofrequency (RF) to smooth fibrillated wrist cartilage surfaces have been reported, most of them concerning the knee joint. In a recent study, Huber *et al*.^46^ examined the thermal risks using RF for the wrist and found that the RF bipolar device could be applied if used with caution since peak temperature in the lunate fossa almost reached 70°C even under continuous irrigation.

#### Bone Marrow Stimulations

No articles about microfracturing of wrist chondral injuries could be found in PubMed, Scholar, or Cochrane, although this surgical technique, according to our knowledge, is used in hand surgery clinics. However, as another bone marrow stimulation technique, Whipple recommended arthroscopic drilling for small chondral defects of 5 mm or less.^47^

#### Juvenile Articular Allografts

Juvenile articular cartilage allografts have been used for treatment of Outerbridge IV^19^ injuries in the knee, ankle, and elbow joints with good outcome.^48-54^ In 2017, a case report showed good result and return to sports with this type of treatment in a wrist osteochondral injury in one patient.^12^ Particulated juvenile articular cartilage (PJAC) allograft has been used to treat Outerbridge Grade IV chondral lesions on the proximal pole of a scaphoid and distal radius scaphoid facet in a patient who had failed conservative management.^12^

#### Autologous Osteochondral Graft

Arthroscopic transplantation of osteochondral autograft from the lateral femoral condyle to distal radius with satisfactory outcome in 4 consecutive patients was reported by Ho *et al*.^13^ In all cases, graft incorporation was complete within 3 to 4 months. All patients showed improvement in wrist function with no pain at follow-up (average of 70.5 month follow-up). Second-look arthroscopy in 3 patients confirmed the preservation of macroscopic normal articular cartilage.^13^ Osteochondral allografts have also been used to treat local cartilage defects of lunate bone. Gaspar *et al*. reported on 2 patients treated by such treatment with good results up to 6 years.^55^

Lee *et al*. reported on one patient treated by osteochondral autograft due to osteochondritis dissecans (OCD) in the scaphoid bone. OCD may have traumatic origin.^56^

#### Chondrocostal Autografts

Chondrocostal grafts have been used and harvested from the ribs, inserted and fixed with plates in place of the articular defect in cases of a malunited intraarticular distal radius fracture (7 cases) or to replace the proximal pole of the scaphoid in cases of SNAC or SLAC (18 cases). In Kienböck’s disease, reports exist of the use of this graft as a free cartilage spacer (4 cases).^57,58^

#### Autologous Chondrocyte Implantations

Autologous chondrocyte implantation is used for failed local cartilage repairs and for large cartilage defects. There is only one case report on successful use of chondrocyte transplantation for the wrist, but no specific results in terms of functional scores, pain, or range of motion were reported in this single case.^59^

#### Bone Resections, Bone Fusions, and Osteotomies

Many techniques exist, but only the most common techniques are described. The HALT chondral injuries (impingement between hamate and lunate) and OA are in 90% of the cases found with concomitant lunotriquetral (LT) ligament injuries. A recommended choice of treatment here is described to be arthroscopic resection of the proximal radial pole of the hamate^4,5^ (**Fig. 9a** and **b**). Degenerative TFCC injuries and ulnar impaction syndrome (impingement between caput ulna and carpus)—see **Figure 2**—are often treated by arthroscopic central TFCC resection and Wafer osteotomy (**Fig. 7**) or in severe cases—with concomitant ulnar positive variance of more than 3 to 4 mm (so-called ulnar impaction)—with open ulnar osteotomy.^7,8^ Ulnar styloid impaction syndrome also exists, with a big ulnar styloid causing conflict, impaction between the tip of the styloid and carpus with focal cartilage injuries at the lunate and triquete.

In SLAC and SNAC I, a radial styloidectomy may relieve the pain and postpone further need for surgery. In SLAC and SNAC II, the most used options are proximal row carpectomy (PRC) or 4-corner fusion (4CF). In the short term, these 2 operations produce similar results with pain relief and an ROM (range of motion) of flexion 30° to 40°, extension 30° to 40°, and 75% maintained grip force.^60-62^

In SLAC and SNAC III (midcarpal arthritis), the only alternative is 4CF or perhaps PRC plus resurfacing of the proximal part of the capitate with RCPI (resurfacing capitate pyrocarbon implant). However, the RCPI implant technique is currently not widely used in the daily clinical routine.^63^

In older patients, with a low ROM preoperatively and a round and blunt-shaped capitate, PRC can be recommended. In patients younger than 35 years or with a pointed, peaked and narrow capitate, 4CF can be recommended. Some skepticism is, however, in order in terms of the long-term viability of a joint with a completely mismatched articular surface between the capitate and the lunate fossa of the radius. Long-term radio-capitate degeneration after more than 10 years of follow-up is seen; however, often asymptomatic and generally only present in about 10% to 20% of patients after PRC.^64^

However, several other studies have reported a significantly larger number of patients with secondary arthritic changes after PRC, although most are symptom free.^65^

There is a lack of well-conducted studies, but Mulford *et al*.^60^ reported and confirmed in a systematic review that both 4CF and PRC produce a clear improvement in pain and subjective outcome measurements for patients with symptomatic SLAC wrists. PRC can perhaps provide a better postoperative ROM, with less risk of the potential complications specific to 4CF (10% more complications occurring; such as nonunion, hardware problems, and dorsal impingement). This systematic review reported that the risk of subsequent OA, albeit most often asymptomatic, is significantly higher after PRC. Subjective outcomes and quality of life, pain relief, motion, and grip strength appear to be similar in both groups.

Radioscapholunate (RSL) fusion could be an alternative in radiocarpal OA after distal radius fracture if the scaphoid and lunate fossae are affected and the midcarpal joint still is intact. Furthermore, when there is complex fragmentation of the articular surface of the distal radius, a silicon foil sheet could be used implanted into the radiocarpal joint to induce a substitute chondral tissue.^66^

With a bone collapse as seen in Kienböck’s and Preiser’s diseases, the cartilage surface is destroyed, and OA has developed. Treatment alternatives are proximal row carpectomy, total wrist arthrodesis, scapho-trapezio-trapezoid arthrodesis, excisional arthroplasty, vascularized bone grafting (VBG), radial shortening osteotomy, radial corrective osteotomy, capitate shortening osteotomy combined with or without VBG, and tendon ball arthroplasty.^41,67^

#### Wrist Denervation

Wrist denervation is regarded to be a safe procedure for the treatment of chronic wrist pain due to OA or ligament instability.^68^

Recently, partial wrist denervation has become popular as a procedure to alleviate chronic pain. However, in a recent review, the authors found improvement in short-term pain relief and functional status but a high re-operation rate. It must be stated, however, that denervation is a treatment of a symptom (pain) and not of the actual cause of pain. Denervation should therefore be used with caution in patients where the root cause of wrist pain is not known, and only after performing adequate preoperative nerve blocks to evaluate the effect of denervation surgery.^69-71^

#### Total Wrist Fusions and Arthroplasties

Total wrist fusion^72^ historically provides predictable pain relief at the cost of a complete loss of motion and shock absorption. The complication rate in total wrist fusion is up to 6%, according to a recent published systematic review.^73^

Total wrist fusion is an option in SLAC and SNAC IV, but total wrist replacement is now also an option, as the survival rate of the new generation of arthroplasties has increased markedly.^17,72,73^

### Retrospective Review of Recorded Wrist Arthroscopies

Due to the disappointment with a low number of reports of cartilage findings after wrist joint trauma, a retrospective analysis of one of the authors’ (JKA) wrist arthroscopies during 1 year was investigated. In the consecutive series (*n* = 48) of wrist arthroscopies (unpublished data), that was evaluated in 2019, 8 cases (16.7%) with chondral lesions were found, often together with concomitant partial or total ligament injuries. Two of them were full-thickness chondral injuries.^19^ Three solitary chondral injuries (2 posttraumatic after intraarticular distal radial fractures and one iatrogenic due to intraarticular screw penetration) were found. The remaining 3 chondral injuries had concomitant partial or total ligament injuries or ulnar impaction syndrome with ulnar positive variance. Ulnar impaction syndrome was treated by Wafer osteotomy^8^ (**Fig. 7**) or converted to open surgery with ulnar shortening osteotomy procedure.^7^ The chondral lesions at the proximal ulnar part of lunate, often seen in conjunction with ulnar impaction were treated by microfracturing (**Fig. 8**) and HALT lesions by shaving and abrasion^4,5^ (**Fig. 9a** and **b**).

The surgeon has the surgical skills level 4 according to Tang and Giddins.^74^

## Discussion

Our literature review concluded that very few articles on cartilage trauma of relevance are present in the current literature and that no consensus can be reached regarding treatment of cartilage injuries in the wrist. Only level of evidence IV-V articles was found. No reports on only isolated cartilage lesions in the wrist were found, rather cartilage lesions were reported in combination with ligament injuries emphasizing the complex interplay between the multiple bones involved in joint motions.^1,29^ About 30% of patients examined with wrist arthroscopies had cartilage lesions findings,^1,29^ while in our reported consecutive series of one year (*n* = 48) of wrist arthroscopies, 16.7% showed cartilage injuries, all related to fractures or ligament injuries.

The difference in incidence may be explained by different patient groups, different diagnostic parameters and different surgical skills, according to Tang and Giddins.^74^ However, the author’s findings that most of the chondral injuries in the wrist have concomitant ligament injuries, is in line with the findings of Lindau *et al*.^1^ and Swart and Tabg.^29^

Posttraumatic OA in the wrist is most often the cause of former ligament injury with gradual development of carpal instability, followed by carpal collapse and degenerative OA (SLAC/SNAC)^3^ (**Fig. 6**) or seen after an intraarticular distal radius fracture. A vascular disruption with subsequent osteonecrosis and OA development as seen in Preiser’s and Kienböck’s diseases might have traumatic origin.

Wrist ligament injuries are, in our clinical experience, often initially neglected as sprains (patient’s and/or doctor’s delay) and with the patients not remembering a former significant trauma, leading to difficulties in associating the initial traumatic injury to the later displayed posttraumatic osteoarthrosis.^75,76^

The wrist is not a weight-bearing joint like the hip, knee, and ankle. Therefore, not all patients suffer from symptoms after wrist cartilage injuries, even if osteoarthritis, such as in SLAC and SNAC has developed.^3^ At the same time, one must keep in mind that the cartilage in the wrist is thin (2 mm) compared with for instance 7 to 8 mm in the patella.^77^ Different anatomical variations in the wrist such as lunate types I or II appear to influence the incidence and localization of chondral injuries.^25,26^ Further studies of different patterns of cartilage injuries compared with anatomical variations in the wrist are needed.

For the development of wrist OA, repetitive microtrauma could be of importance. Jones *et al*.^78^ examined the prevalence of hand and wrist OA in elite former cricket and rugby union players and found that former elite cricketers reported more hand pain than rugby players and speculated that risk factors aside from injury, such as chronic load or repetitive microtrauma, may be more prominent in the development and progression of hand and wrist OA in former elite male athletes.

There are several limitations to this study. No follow-up or functional scores in terms of the author’s (JKA) recorded wrist arthroscopy cases were used. There is a risk of selection bias in the small series of wrist arthroscopies performed. There is also always a risk of publication bias in articles with small sample sizes. The scarce numbers of available articles limited the results and conclusions. The articles found in the literature review were case reports (LoE V) or case series (LoE IV) with few included patients with short term follow-ups. Only English written articles were included in the literature review which is a limitation related to existing papers published in other languages describing also wrist injuries.

However, the techniques of wrist arthroscopy were developed throughout the early 1980s. At first, arthroscopy of the wrist was an innovative diagnostics, but a few years later, wrist arthroscopy became increasingly used also as a therapeutic tool.^79-85^

Cartilaginous lesions in the wrist may be fairly common.^1,29,36^ Ulnar impaction, degenerative LT injuries, and HALT are often seen with concomitant cartilage lesions as a sign of prior trauma affecting the whole ulnar column of the wrist.^4,7^ Diagnosis of cartilage lesions in the wrist remains difficult,^21^ and the determination of when to operate is not at all clear. However, as wrist OA is a common finding in middle-aged persons indicating that more knowledge is needed to determine better the fate of isolated cartilage lesions related to progression into wrist OA. Careful attention to the medical history and physical examination can make cartilage lesion diagnosis easier, and appropriate surgical intervention can be useful in alleviating symptoms and returning the patients to normal activities as well as being joint protective. Although short-term results are promising, long-term studies are needed before the efficacy of arthroscopic management of cartilage lesions of the wrist is known.^18,20,59,85^

Larger prospective comparative studies are needed to get any further ideas about the real incidence of chondral injuries in the wrist, their localizations, and severity, the outcome after surgery^20^ as well as comparative studies to learn which chondral repair techniques may be recommended in the different types of injuries. A limiting factor, however, is that the number of wrist arthroscopic procedures is inferior to knee and shoulder arthroscopies. However, perhaps a noninferiority study^86^—comparing microfracturing with more novel techniques—or multicenter study could be possible to perform in the future.

## Conclusion

There is a lack of knowledge in current literature on cartilage and osteochondral injuries, as well as posttraumatic OA in the wrist. There is also a lack of knowledge with regard to treatment of these injuries. Cartilage injuries appear to be rather common, being found in 17% to 32% of all wrist arthroscopies after trauma, but no guidelines regarding conservative or surgical treatment can be recommended at the moment. Larger prospective comparative studies, noninferiority studies, or multicenter studies are needed.
